# Protecting peatlands requires understanding stakeholder perceptions and relational values: A case study of peatlands in the Yorkshire Dales

**DOI:** 10.1007/s13280-023-01850-3

**Published:** 2023-04-23

**Authors:** Kirsten J. Lees, Rachel Carmenta, Ian Condliffe, Anne Gray, Lyndon Marquis, Timothy M. Lenton

**Affiliations:** 1 University of Derby, Kedleston Road, Derby, DE22 1GB England; 2 Tyndall Centre, University of East Anglia, Norwich Research Park, NR4 7TJ Norwich, United Kingdom; 3 Independent, Ilkley, United Kingdom; 4 The Heather Trust, DG1 2RL Dumfries, United Kingdom; 5 Yorkshire Peat Partnership, BD23 1UD Skipton, United Kingdom; 6 The Global Systems Institute, University of Exeter, EX4 4QE Exeter, United Kingdom

**Keywords:** Moorland, Nature-based solutions, Peatland, Plural values, Q-method, Relational values

## Abstract

Sustainable peatland management is a global environmental governance challenge given peat’s carbon storage. Peatlands worldwide are sites of contested demands between stakeholders with distinct management priorities. In the United Kingdom, peatland management is a focus of political interest for nature-based solutions (NBS), causing tensions with land managers who feel their traditional knowledge is undervalued. Using Q-method (a semi-quantitative method for clarifying distinct viewpoints) with estate managers, gamekeepers, farmers, and employees of land-owning organisations, we explored perceptions around changing upland management in the Yorkshire Dales. Land managers hold strong values of ownership, aesthetics, and stewardship. The prospect of changing management causes fears of losing these relational values alongside instrumental values. Yorkshire Dales stakeholders agreed on NBS aims (reducing flooding, limiting wildfires, protecting wild birds), but disagreed on methods to achieve these. Our research supports engaging local stakeholders at all stages of peatland protection schemes to minimise resentment towards top-down management.

**Supplementary Information:**

The online version contains supplementary material available at 10.1007/s13280-023-01850-3.

## Introduction

Peatlands are increasingly recognised as landscapes of global significance, due to their influence on the long-term global carbon cycle (Loisel et al. 2020) and ecosystem functions including hydrology and biodiversity (Martin-Ortega et al. 2014; Law et al. 2015; Minayeva et al. 2017). Despite their significance, peatlands are globally under threat from land use change, drainage, degradation, or removal due to a variety of anthropogenic pressures (Rawlins and Morrs 2010; Turetsky et al. 2015; Ribeiro et al. 2020), and there is now increasing focus on reorienting peatland management in recognition of their crucial role in providing nature-based solutions (NBS) to climate change impacts (Strack et al. 2022). Peatlands store substantial amounts of carbon (Yu et al. 2010), but drying and damage cause carbon losses as organic material is oxidised (Liu et al. 2016). Rewetting damaged peatlands can therefore protect carbon stores and encourage healthy functioning of the peatland carbon cycle, thereby increasing carbon uptake and storage (Nugent et al. 2018). Peatlands are also valued for their diverse economic and cultural services, which may require intensive land management to maintain (Byg et al. 2017). The tension between management of peatlands for climate mitigation and sustainability goals versus numerous alternative potential outcomes (such as plantations, grazing, field sports) results in often contested landscapes (Carmenta et al 2017; Davies et al. 2016; Goldstein 2016). From the tropics to the UK uplands, configuring more sustainable, and equitable, peatland management is a climate-significant governance challenge that will require understanding the divergent viewpoints operating in peat landscapes and their interplay on the ways in which peat landscape management impacts people–nature relations (Zafra-Calvo et al. 2020).

IPBES (Intergovernmental Science-Policy Platform on Biodiversity and Ecosystem Services) recognises three types of people–nature relations in the Nature Futures Framework. These are instrumental (nature for society), relational (culture embodied and enabled by nature) and intrinsic (nature for nature) (Diaz et al. 2015; Pascual et al. 2017). Environmental governance decisions impact these relations and thereby can create enablers and barriers for stakeholders to engage in, support, or reject management strategies. For instance, barriers may derive from anticipated (or experienced) impacts to instrumental relations, affecting the material use and economic benefits stakeholders are able to gain from the land (Rawlins and Morrs 2010). Barriers may also arise from impacts to relational values, such as impairing place attachments or identities derived from place (Kibler et al. 2018; Urquhart and Acott 2014; Chan et al. 2012; Mould et al. 2020). (Note: throughout this article, we use the word ‘stakeholder’ to refer to those who are affected by or can affect a decision or issues, whilst acknowledging that the term has been used in negative ways in the past, following Reed 2022).

We were particularly interested in the relational values held by stakeholders with regard to proposed changes to peatland management in the English uplands, away from diverse traditional management using drainage and managed burns for grouse moors and livestock, towards restorative NBS approaches to enhance the water and climate services of the landscape. Relational values have ben understudied in peatlands, but are likely to play a significant role in the contested natures of these landscapes (Daeli et al. 2021; Flood et al. 2021). Relational values encompass emotive constituents related to connection to a place, and the contribution of the landscape to enabling particular personal and community activities that generate meaningful identities and social relations. In the conservation community, relational values have routinely been omitted from understanding the contributions of place to people, and their salience is now increasingly recognised as vital to enabling shifts to more sustainable stewardship (Chan et al. 2018).

In this study, we used Q-method, a semi-quantitative method (i.e. combines both quantitative and qualitative analysis), to give insight into the shared subjectivities and perceptions of stakeholders in relation to management options for a peat-dominated national park in the UK (Eden et al. 2005). Q-method is particularly useful for identifying the perceptions of disparate stakeholders on complex and contested issues. It organises the complexity of particular viewpoints in a holistic approach that identifies how particular constellations (or bundles) of individual perceptions combine to generate an emerging and holistic viewpoint (i.e. rather than attempting to reduce this complexity into individual opinion statements) (Zabala et al. 2018).

### Case study

Upland peatlands in England today are working landscapes where livestock grazing and field sports (and historically peat cutting) have traditionally played a considerable role in the management and appearance of the landscape (Soliva et al. 2008; Lees et al. 2021). However, alternative management practices focused towards climate and conservation goals are now becoming encouraged through government-supported incentives, including considerable interest in the potential of peatlands to contribute towards NBS to climate change impacts. NBS-related practices in peatlands consist of blocking drainage ditches to raise water levels, and employing interventions that aim to increase natural vegetation cover (i.e. *Sphagnum*, cotton grass (*Eriophorum angustifolium*), etc.) and alter the mix of species from bare ground or monocultures to more mixed communities (Thom et al. 2019). These sets of interventions are often referred to as peatland restoration, or ‘managing peatlands for carbon and water’ (the latter is the term applied throughout this study, shortened to MPCW). The term ‘restoration’ can be controversial, with critics stating that the ‘natural’ state of a peatland area might be woodland (IUCN UK Peatland Programme 2020), which is not something that most restoration schemes aim for (Thom et al. 2019). Although perceived as normatively ‘good’ practices by conservationists, these interventions do not always meet approval with all land users and in some cases infringe on place-based understandings of how peatland should be managed (Heather Trust 2019). For example, some land managers assert that MPCW will be detrimental to farm livestock output, or the viability of sustaining grouse populations important to the field sports sector. Others are concerned that MPCW will increase the risks of large wildfires on peatlands (Davies et al. 2016).

The contested views about managing peatlands have been presented by the media as a simple two-sided debate between traditional use and MPCW (Davies et al. 2016; Heather Trust 2019). Conservation activists often play into this narrative by vilifying all traditional management practices, which in turn has created some resentment amongst traditional land managers who defend their practices (e.g. Nolan 2021). However, as peatland management occurs on landscapes of multiple diverse benefits to multiple stakeholders, there is a need to better understand these different perceptions and attempt to reconcile them towards shared understanding. Previous studies have used scenario development to explore UK upland futures (Reed et al. 2013); in this study, we build on this work by exploring in more depth a single scenario and the diverse values and perceptions associated with it. Such analysis and research can serve as a boundary object from which to have more nuanced conversations about management possibilities, and the distribution of impacts across groups.

## Materials and methods

### Study area

**Fig. 1:**
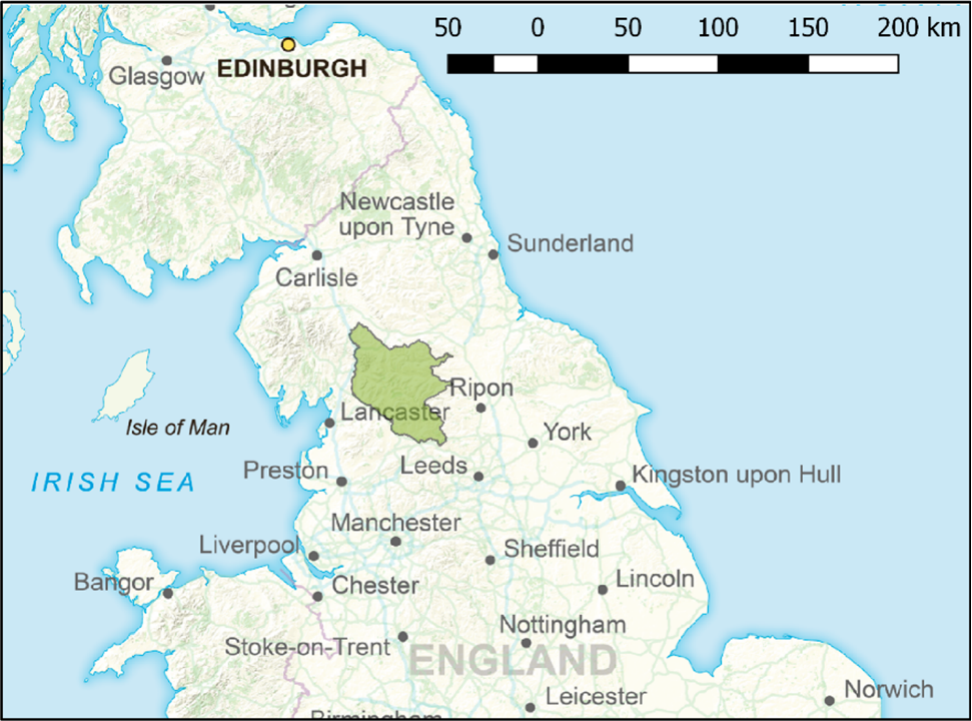
Location of Yorkshire Dales National Park in northern England. Contains OS data © Crown copyright and database right 2022

The Yorkshire Dales is one of the UK’s fifteen National Parks and is situated in the North of England (Fig. 1). The peatland area within the Park is 58 545 ha with a mean depth of 1.10 m and is mostly heather-dominated (*Calluna vulgaris*) blanket bog, although there is a change to M2, M3, M17, and M18 NVC (National Vegetation Classification) communities where restoration is starting to take hold. The landscape is largely managed for livestock farming and grouse moor management. However, interest and investment aimed at restoring peatland are growing (UK govt 2021). At the time of publishing, Yorkshire Peat Partnership (YPP) has worked on 22 971 ha of peatland within the National Park, usually beginning with work to restore hydrological function to areas of dried peat, before revegetating damaged surfaces (see photos in Box 1). YPP’s widespread work meant that land managers who participated in the study were likely to have some knowledge of these schemes and perceptions about their impacts. YPP is in the process of deploying IUCN UK Peatland Programme’s Eyes on the Bog methodology alongside GHG emissions monitoring to give important information about the impacts of this work. It is likely that the results will be similar to those in other areas in terms of reducing flooding and limiting carbon emissions (e.g. Dixon et al. 2014; Shuttleworth et al. 2019).

### Q-method

Q-method seeks to identify the commonalities between participants rather than correlations between perceptions (Eden et al. 2005). It is designed for use with a small but diverse sample of participants and is therefore suitable for a subject area where stakeholders are a relatively small group.

#### Designing the Q-set

Q-method involves asking participants to sort and rank a set of statements (the ‘Q-set’) in a forced normal distribution along a scale; in our case, the scale was from ‘most’ to ‘least important to me’. The statements composing the Q-set should represent the broad range of diverse opinions on the matter of study. In order to ensure this breadth, statement generation was informed by literature review and particularly drew upon the discussion series ‘What are Britain’s Uplands for?’, which held conversations with land managers, conservationists, and other interested parties around Britain, including in English peatland areas (Heather Trust 2019). Statements were further developed in a one-day workshop (held in Skipton) with all project partners and were additionally adapted following piloting with three peatland land managers. The final Q-set comprised 54 unique statements (see Supplementary Information).

**Box 1:** Scenario statement given to participants, and images of peatland before and after management interventions

**Managing peatland for carbon and water (MPCW)** Managing peatland for carbon and water aims to restore the peatland to a state in which carbon is taken in by the vegetation and stored as it decomposes into peat, and rainwater moves slowly through the landscape and is naturally filtered before reaching watercourses. The ideal peatland under this scenario has high water levels all year round, and a mix of vegetation including *Sphagnum* moss, dwarf shrubs such as heather, and sedges. The peat surface should be wet enough that kneeling or sitting on it gets clothes wet immediately. Managing peatland for carbon and water involves a variety of approaches. These can include blocking grips and gullies to raise the water table, leaky dams on watercourses to slow run-off, stabilising and revegetating bare peat to reduce erosion, and altering the mix of plants present. Frequent burning is unlikely to be compatible with managing for carbon and water, although heather cutting may be. Grazing can be used as a management method to limit dominance of a single vegetation species, and should be at stocking densities which allow healthy mixed vegetation to grow. *Photos show East Gill in Nidderdale (an AONB adjacent to the Yorkshire Dales National Park) taken a year apart. Left: image before restoration (credit YPP/Jenny Sharman); Right: image a year after restoration interventions, showing coir logs added to stabilise the surface and retain water (credit YPP/Aaron de Raat).*	

To aid in implementing the Q-method, a scenario statement (see Box 1) was developed intended to generate a systematised understanding of what was meant by ‘managing peatland for carbon and water’ (MPCW). The statement was designed to convey the *de jure* plan for what this management would entail (Thom et al. 2019). This scenario was developed alongside the Q-set, in consultation with YPP which is involved in the management of peatland for these objectives. Participants were asked to read the scenario statement and then sort the Q-set in response to it.

### Participant recruitment

Participants were recruited through a combination of snow-ball sampling and advertisement through The Heather Trust, Yorkshire Dales Moorland Group, Moorland Farmers, and GWCT (Game & Wildlife Conservation Trust). Snow balls were started through the membership and networks of the project partner organisations (i.e. The Heather Trust and YPP).

We specifically recruited participants with responsibilities for land management decisions in this study (i.e. not visitors or local communities with no land management responsibility). We made this decision to study the perceptions of management methods and consequences, and associated values, in greater depth; although visitors and local communities will hold opinions and preferences, they may not be aware of the complexities of the ecosystem processes or the details of peatland management. Furthermore, the project partners were most interested in understanding why certain stakeholders are reluctant to engage with MPCW, and we therefore targeted groups with relevant decision-making responsibilities. As part of the process, we asked participants about their previous engagement with MPCW. The majority of participants had been actively involved in rewetting schemes (*n* = 6), whilst some others were aware of its occurrence at other sites (*n* = 2). The final sample (p-set) included estate managers (*n* = 2), employees of land-owning organisations (*n* = 2), gamekeepers (*n* = 3), and farmers (*n* = 2). We specified that the individuals should complete the method based on their own personal views rather than what their organisation would prioritise.

### Implementing the method

We successfully adapted the in-person interview process using posted participant packs (including the scenario and statement set and the placement grid) and videoconferencing (× 2 calls via Zoom) in the wake of the Covid-19 pandemic (see Supplementary Information).

In the first call, the researcher introduced themselves, the project, and the free-prior and informed consent process. The participant was asked to read the statements and make an initial sort into two bins: ‘important to me’ and ‘unimportant to me’ before the next call. We developed a standardised operating procedure (SOP) which involved explaining that the scenario was developed to reflect policy changes, whilst the statements were perceptions rather than scientific facts (see Supplementary Information). In the second call, the participant sorted the statements onto the grid whilst discussing their placement with the researcher. In some cases (*n* = 2), the participant had already completed this exercise before the call, and the researcher then asked about the placement of the five statements in the most extreme positions (i.e. most important, and least important). Interview notes were made to support the interpretation of the statement placements in the analysis. The first interviews lasted approximately 15 min on average, and the second approximately 45 min.

Each participant was thanked for their time with a gift voucher and received a short report on the results.

### Statistical analysis and interpretation

**Fig. 2:**
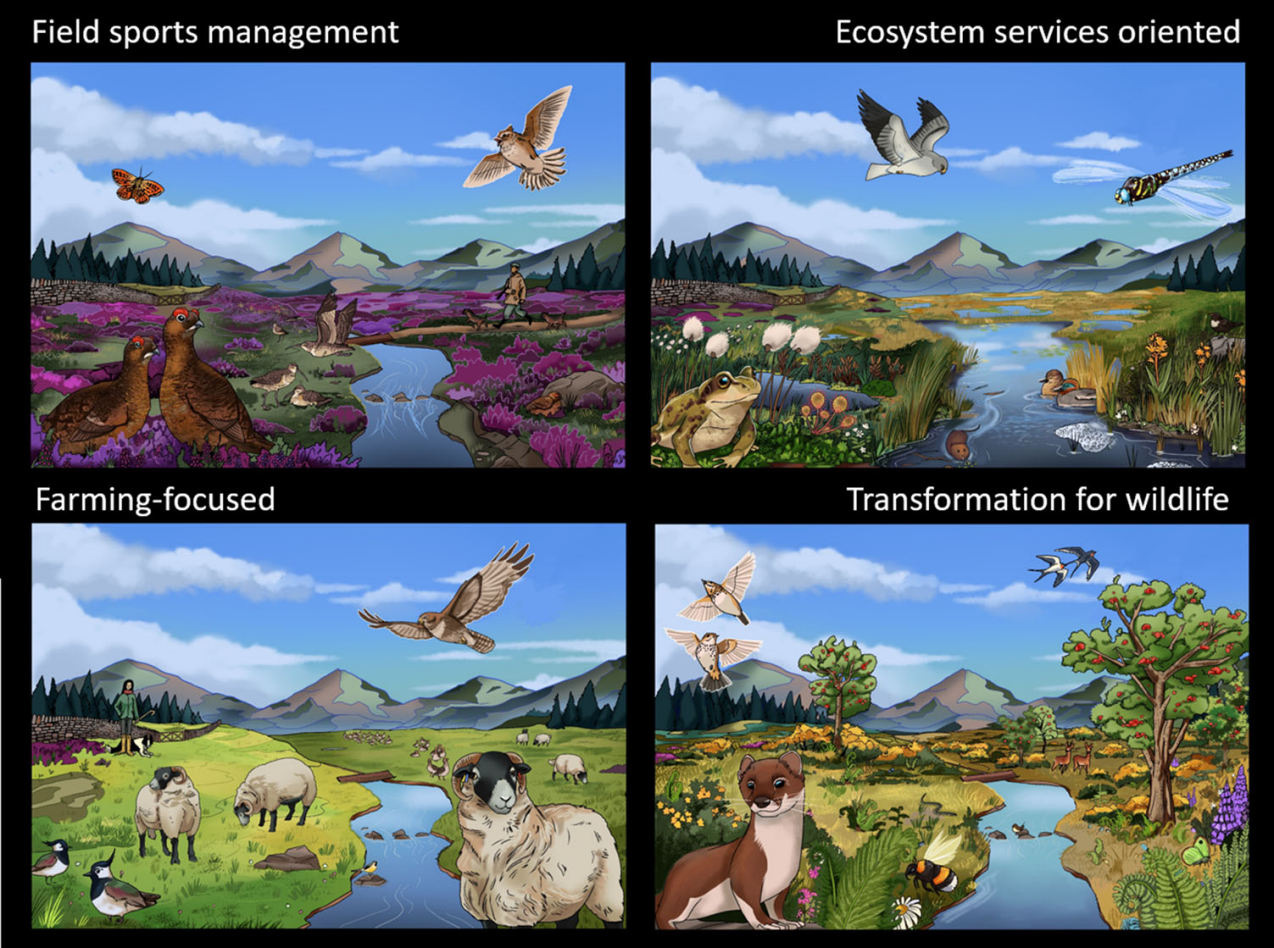
Artist’s impressions of what a peatland area could look like in the future under the different priorities of the four viewpoints (note that these images are extreme examples for illustrative purposes, in reality there would likely be some overlap between them)

The Q-sorts were analysed using the Q-method package in R (Zabala 2014), which applies principal component analysis (PCA) to the data; three factors (i.e. viewpoints) were initially extracted. Factors were interpreted through considering a combination of the factor loadings for each statement, and the interview notes, which is the semi-quantitative nature of the method. This process led to the recognition that Factor 2 was significantly positively loaded with two participants, but significantly negatively loaded with a third, suggesting that Factor 2 actually represents two viewpoints in opposition. Factor 2 was therefore split into Factor 2 and Factor 3. Together, these four factors explained 60% of the variance of the sample. In the discussion below, we refer to participants who were significantly loaded onto a factor as being ‘strongly associated’ with it, and those who also had a clear but non-significant loading onto a second factor as being ‘weakly associated’ with the second factor. Once the four viewpoints were defined and interpreted, we worked with a visual artist to translate them into images (see Fig. 2). The purpose of these visuals is to show alternative futures. Having visual representations of these potential futures allows people to imagine and therefore work towards desired states (Hicks 1996) and is also a good starting point for discussion and debate (including whether or not such outcomes are likely).

We assessed factors to look at emergent themes, including attributes in common and those held in contention. We were particularly interested in drawing out the relational values of stakeholders. To do this, we completed thematic analysis of participant quotes, which brought out four main relational values for further discussion.

## Results

Semi-quantitative analysis of our results distinguished four viewpoints (Fig. 2): traditional management for field sports (‘Field sports management’, Factor 1) representing the views of four out of five estate managers and gamekeepers; improve water quality, limit flooding, and reduce erosion (‘Ecosystem services oriented’, Factor 2) strongly associated with the two employees of land-owning organisations; a farming-associated viewpoint focused on land management autonomy and income concerns, strongly associated with one farmer (‘Farming-focused’, Factor 3); and finally a focus on sustainable, wildlife friendly long-term management (‘Transformation for wildlife’, Factor 4) which was associated with a mixed group of stakeholders (Table 1).

**Table 1:** The four factors explained, with key quotes selected through qualitative interpretation to illustrate defining values and beliefs. The participants’ strongest factor associations are given in bold. Weaker associations are also shown in italics

Factor	Participants associated with factor	Description	Key quotes
Field sports management (Factor 1)	**2 estate managers** **2 gamekeepers**	Wants to maintain current management regimes including burning and predator control Expresses local knowledge of ecosystem complexity Asserts interfering with the current management of peatlands would create burdens for incomes, identities, bird populations MPCW would increase wildfires Recognises benefits of rewetting for limiting summer droughts and increasing crane flies to feed grouse and other wild birds Concerns about heather beetle	‘[Grouse moor management is] the only source of income underpinning safe management of blanket bog’ ~ Estate manager ‘if something didn’t add up I’d be out of a job and out of a home’ ~ Gamekeeper ‘[letting driven grouse shooting become economically unviable] strikes at the heart of my personal view’ ~ Estate manager ‘where there is not burning there’s an increased risk of dangerous wildfire’ ~ Estate manager ‘in warmer, drier summers these rewetted areas—they’re a stronghold for these insects’ ~ Gamekeeper ‘If you'd managed the moor as long as I have and seen the moor die [due to heather beetle damage]- you would cry’ ~ Gamekeeper
Ecosystem services oriented (Factor 2)	**2 employees of land-owning organisations** *1 gamekeeper* *1 estate manager*	MPCW to limit flooding and improve water supply MPCW to increase vegetation diversity and reduce erosion Livestock farming considered unsuitable for peatlands	‘those three I’d grouped together, all around natural flood management, water quality stuff’ ~ Organisation employee ‘[Clean water provision is] hugely important from the public perspective’ ~ Organisation employee ‘[Heather dominance] drives the peat out’ ~ Organisation employee ‘[increasing vegetation cover] links with reducing soil erosion’ ~ Organisation employee ‘shouldn’t have livestock on blanket bog anyway’ ~ Organisation employee
Farming-focused (Factor 3)	**1 farmer**	Wants greater autonomy in landscape management decisions Considers MPCW schemes disregard past efforts to improve the land and ignore potential future problems The issue of control was also associated with concerns about deleterious impacts on income Prioritises livestock related burdens such as liver fluke and bog asphodel	‘might as well be working for someone else’ ~ Farmer ‘[to] all of a sudden get told to backtrack—[it’s] really kicking someone in the teeth’ ~ Farmer ‘[You think]’that won’t work’, but you have to do it anyway’ ~ Farmer ‘[I’ve] never read as much government stuff, trying to see what they’re thinking’ ~ Farmer
Transformation for wildlife (Factor 4)	**1 farmer** **1 gamekeeper** *1 gamekeeper* *1 organisation employee*	Emphasises pride, custodianship and management prioritising wildlife and nature Highlights that change is needed, even if not always wanted (predicts end of grouse shooting) Focuses on the longer term, and vision of sustainability	‘I do like to see…things like ring ouzels’ ~ Gamekeeper ‘doing it as a matter of pride to make the farm a better place’ ~ Farmer ‘let nature go—it’d be lovely’ ~ Organisation employee ‘I can see grouse shooting not being here in ten years’ ~ Gamekeeper ‘has to work on a long time scale—have to make sure it’s managed for the long term’ ~ Organisation employee

**Fig. 3:**
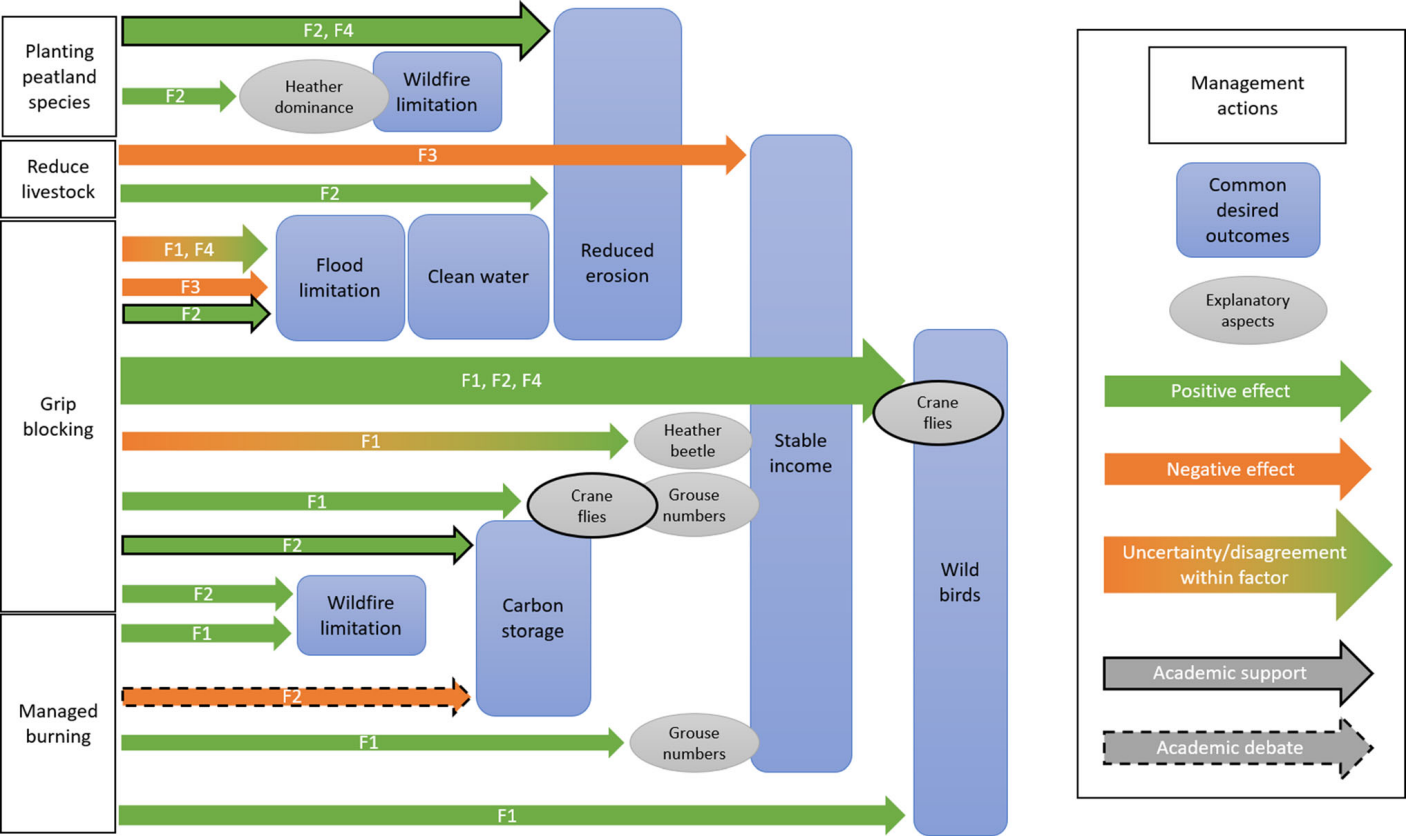
MPCW involves particular management actions, and different viewpoints perceive their consequences distinctly. This figure also shows the degree to which a perception is supported by academic evidence or is subject to debate. Perceptions without either support or debate are either discredited by the majority of academic work (in these cases the opposing view is shown on the diagram as having academic support), or represent a knowledge gap. The explanatory aspects are specific to the arrow directed to them, and help to explain the relationship between the management action and the desired outcome

Interestingly, these four viewpoints converged around their shared desire for similar outcomes of peatland management: reduced flooding and wildfires, stable incomes, and improved wild (as opposed to managed grouse) bird numbers. Several of the statements reflecting the regulating ecosystem services provided by peatlands, such as clean water, reduced erosion, and carbon storage, were rated as relatively important (> 0) by all three factors emerging from the initial principal component analysis. These areas of consensus may be suitable anchor points for peatland management discussions. However, beliefs about the ability of different management methods to achieve these desired outcomes (Fig. 3) were distinct (explored in “Conservation methods, not aims, are the source of disagreement” section).

We found that there were several beliefs and values held in common across more than one factor, such as an appreciation of wild birdlife (Table 2). Our thematic analysis of participant quotes brought out four main relational values (Table 2), three of which were common across all factors: sense of ownership, aesthetics, and stewardship. The fourth, fear of loss or wasted resources, was specific to the field sports management (F1) and farming-focused viewpoints (F3).

**Table 2:** Beliefs and values held in common by more than one factor, with illustrative quotes. This includes the four relational values that emerged from our thematic analysis

Beliefs and values held in common	Associated factors	Description	Key quotes
Sense of ownership	All factors	Feeling of ownership even when not landowners Related to long-term engagement with the land Exclusion of ‘others’	‘we've managed these uplands for decades—now there's people trying to interfere’ ~ Gamekeeper ‘[we] farm in an old-fashioned way to make sure we live in a beautiful place’ ~ Farmer ‘[I] don’t want them [tourists] on my moors’ ~ Organisation employee
Aesthetics	All factors (but aesthetic preferences varied between different stakeholders)	Reluctance to give aesthetic preferences when asked directly Appreciation of existing landscape Ambivalence/negativity towards specific local changes in land appearance Positive visions for the future	‘beauty is in the eye of the beholder’ ~ Gamekeeper ‘I don’t think it's possible to create a more beautiful landscape than we already have’ ~ Gamekeeper ‘[I was] bemused by all the grips blocked to 0.5 m above ground level—altered the whole aspect of the moor’ ~ Gamekeeper ‘[a fell planted with trees] doesn’t half look a mess’ ~ Farmer ‘looks better when it’s all green and nice’ ~ Farmer
Stewardship	All factors (but interpretation of the ‘right’ management varied between different stakeholders)	Sense of duty and legacy Personal vindication/validation Custodianship rather than possession	‘[We are] farming to keep it right’ ~ Farmer ‘[We want to] leave it better than we acquired it’ ~ Farmer ‘[I’m] not that bothered if people don’t agree—as long as we know we're doing the right thing’ ~ Organisation employee ‘The older you get the more you realise you're just a custodian’ ~ Gamekeeper
Fear of loss or wasted resources	Field sports management Farming-focused	Fear of losing personal joys associated with the land Fear of losing identity Fear of change causing damage Fear of using the land in a ‘wasteful’ way	‘[I] would be sad not to [hear the curlews]’ ~ Estate manager ‘I don’t want to lose sight of that we are farmers’ ~ Farmer ‘heather beetle, dams bursting—the worry of that’ ~ Gamekeeper ‘planting trees on good land—that's terrible…all that dry land could be used for production of something—even biomass would be more use’ ~ Farmer
Dislike of top-down management approach	Field sports management Farming-focused Transformation for wildlife	Loss of control Decision-makers are incompetent Agendas change rapidly and don’t consider long-term consequences Each area requires different management	‘[You think]’that won’t work’, but you have to do it anyway’ ~ Farmer ‘institutional bias against people’ ‘practitioner has no value’ ~ Estate manager ‘working relationship [with NE is] at an all-time low’ ~ Gamekeeper ‘huge amounts of public money spent on projects trying to tick as many boxes as possible, not looking into the future’ ~ Gamekeeper ‘nature isn’t black and white’ ~ Estate manager ‘people know their land’ ~ Farmer
A full sponge causes run-off	Field sports management Farming-focused	Belief that organisations promoting rewetting aim to reduce floods by increasing water storage in the peat (the peatland sponge metaphor) Rewetting may actually increase flash flooding and erosion when dams burst	‘once the sponge is full, they seem to think it’s going to hold more’ ~ Farmer ‘if you block everything up…once a sponge is full all the water comes out’ ~ Gamekeeper ‘we are holding back more now than ever was’ ~ Gamekeeper ‘seen a big increase in local flash flooding’ ‘can’t pin it down to rewetting, but doing our homework…’ ~ Gamekeeper
Importance of wild birdlife	All factors	Personal appreciation of seeing and hearing the wild birds Belief that favoured management style is better for wild birds than others	‘I do like to see…things like ring ouzels’ ~ Gamekeeper ‘[Management is] not just [for] the grouse—for all the birds that live on the moorland’ ~ Gamekeeper

Some of the statements were considered unimportant to decision making (rated < 1) by all of the factors (see SSupplementary Information, Table S1). For instance, livestock drownings and vehicle bogging were considered unrelated to MPCW interventions. Nature tourism was widely disregarded either as an unlikely source of income, or as having negative consequences for the environment. There was much less concern around protection of heritage through traditional management than we expected, as most participants recognised that the Yorkshire Dales have always been landscapes of change. It is worthwhile to recognise which issues were not important, as these may be candidates of less priority in the future dialogue around peatland management.

## Discussion

Our analysis identified four distinct viewpoints (Fig. 2 and Table 1) concerning managing peatlands for carbon and water. The first **Field sports management** viewpoint represented the views of the majority of estate managers and gamekeepers in the study and favoured maintaining traditional management methods, especially regular burning to promote heather growth. Traditional management was considered best for birds and for limiting wildfires and to provide an income for local communities. It favoured changing management where clear benefits could accrue, for example, wetter areas supporting insects to feed chicks.

The **Ecosystem services oriented** viewpoint—most strongly associated with employees of land-owning organisations—prioritised management for ecosystem services outcomes such as reduced erosion, mixed peatland vegetation communities, and clean water. This was therefore most highly aligned with the MPCW aims.

The **Farming-focused viewpoint** (most strongly associated with a farmer) was most concerned with retaining control over the land, autonomy in decision making, and the effects of MPCW on livestock.

The **Transformation for wildlife** viewpoint, composed of multiple stakeholder types (including gamekeepers, farmers, and employees), wanted management to prioritise nature and wildlife rather than human uses. There was a belief within this viewpoint that dramatic changes in upland peatlands were necessary or inevitable. This viewpoint was also the most concerned with the long-term impacts of management, and whether they would be sustainable into the future.

It is important to note that the small sample size of the study (partly due to Covid-19 restrictions at the time) means that these factor divisions may not be reliable in application to a wider community of stakeholders, and should be considered an intermediate result encouraging further research in this area.

The following discussion sections (“Conservation methods, not aims, are the source of disagreement”–“Problems with the top-down approach to land management”) focus on the areas of alignment and contrast between the viewpoints.

### Conservation methods, not aims, are the source of disagreement

Many of the participants expressed similar desired outcomes, but the methods for achieving those outcomes differed greatly (Fig. 3). An example of this is wildfire limitation. All participants desired to see fewer wildfires, and all agreed that management methods to minimise the damage caused by such events should be promoted. However, people associated with the field sports management factor believed that the best method to employ for this purpose was managed burning, as this reduces the amount of out-of-control ‘rank’ heather. People associated with the ecosystem services-oriented factor believed that the best method was instead to reduce heather dominance and encourage a mix of vegetation that would hold moisture in the peat (Fig. 2). Disagreement was therefore not necessarily in what people wanted from the peatlands, but rather the best way to bring about those outcomes. This is an important result for those who seek to encourage peatland management for carbon and water, because it indicates that convincingly explaining and co-creating the rationale for these changes is at least as important as the ultimate end goals.

Another example of agreement on aims but disagreement on methods is flood limitation. This is a key ecosystem service promoted by conservation groups, but misplaced analogies contribute to conflict with land managers. The metaphor comparing peatland to sponges that can hold large amounts of water is widespread, and is often interpreted to mean that restoration will increase the water held in the peat (like an expanding sponge), thereby limiting flooding. When managers can see that their land is already wet and at maximum capacity (especially in the winter when flooding is more likely), it seems obvious that it cannot hold any more water (‘the sponge is full’, see Table 2). Communication using the peatland sponge metaphor to explain flood limitation is therefore seen as out-of-touch with reality, and the whole concept of flood limitation through MPCW is rejected (see Table 2). The current understanding is that although the peat itself cannot expand indefinitely to hold unlimited water, a healthy vegetated peatland surface can still slow run-off and thereby limit flooding (Shuttleworth et al. 2019). A new metaphor for peatland flood limitation that better illustrates this is urgently needed.

Statement 27 ‘Increase carbon capture to help meet net zero by 2050’ did not generate extreme rankings in any of the viewpoints. It was placed at the ends of the grid by two participants (rated as 5 and -4 by two different employees of land-owning organisations), but these participants effectively cancelled each other out in the ecosystem services viewpoint, meaning that carbon was not considered important in this factor. This is likely to be a situation where a single individual with an unusual perception had a disproportionate impact on the overall factor due to the small sample size. There was some evidence that carbon is slowly increasing in importance within the other viewpoints, for example, one gamekeeper said 'until we started this work [restoration on the estate] I didn’t understand it [the importance of peatlands for carbon]’. Another gamekeeper said ‘I'd like to see evidence that it is actually working’. Generally, therefore, we can suggest that carbon is not a major factor in decision making for many stakeholders, but providing education and evidence may increase its importance.

Much of the disagreement on methods was due to conflicting understandings of ecosystem processes and feedbacks (Fig. 3). As one estate manager said, ‘nature isn’t black and white’. Many participants felt that the complexities of peatland ecosystems mean that different management styles may be best for different areas of land. One farmer responded to S25 ‘Need to involve external experts to plan management’ by saying ‘every spot’s different’, and reiterated at multiple points during the conversation that ‘people know their land’. MPCW is complex, as the different management interventions recommended to achieve this aim each have a range of consequences and interactions with other options. Selecting a suitable toolset of interventions for each land area requires discussion between restoration practitioners and local land managers. This understanding of the variations within a landscape suggests that policy practices encouraging specific management actions do not work as blanket guidelines.

### Areas where factors were in opposition

Areas of opposition are important in defining the differences between factors. In some cases, these oppositions are obvious, as in the case of the ecosystem services-oriented factor 2 and farming-focused factor 3. The ecosystem services-oriented factor rated statements concerning water quality and flood limitation very highly, whilst the farming-focused factor did not. One farmer said they were a ‘believer in dredging’—‘rivers fill with gravel all the time’—‘[it’s] keeping on top of a job’. They explained that they have a digger to pull gravel out of the beck, which keeps it in the same path: ‘keeps it right’. This is clearly in opposition to the ecosystem services-oriented factor which was in favour of rewetting and more natural flood management methods. We note, however, that it was particularly difficult to recruit farmers for this study, perhaps because tenant farmers felt that they did not fit the ‘land manager’ description. A larger study of farmers might have more diversity of viewpoints. Participants associated with the ecosystem services-oriented factor rated farming concerns as unimportant and expressed the opinion that we ‘shouldn’t have livestock on blanket bog anyway’ (organisation employee). This opposition between the ecosystem services-oriented and farming-focused factors is linked to the more general disagreement on methods, but agreement on aims (see Sect. "Conservation methods, not aims, are the source of disagreement") as both factors were keen to limit flooding, but promoted methods that were in opposition.

There was also some opposition between the field sports management factor and the farming-focused factor. Generally, the participants associated with the field sports management factor rated concerns about livestock farming as unimportant: [S4: Livestock drowning in wetter areas] ‘doesn’t bother me that much’ (gamekeeper). On the other side, those associated with the farming-focused factor scored grouse-shooting concerns as unimportant: ‘[I’m] not a believer in grouse shooting’ (farmer). These two factors were similar, however, in their high ratings for income sources and issues of landscape control.

The ecosystem services-oriented and transformation for wildlife factors had a correlation of 0.25, which suggests a level of agreement between the two factors. Differences were evident, however, in the motivations for changing management. The ecosystem services-oriented factor was in favour of change for ecosystem service outcomes, whereas the transformation for wildlife was more concerned with aspects such as aesthetics and wildlife. This contrast reflects the ongoing debate between those who promote neoliberal conservation (attaching economic value to natural systems) and those who promote an intrinsic worth (nature for nature) (Wyborn et al. 2020; Apostolopoulou et al. 2021).

### Science and peatlands

The role of scientific research and debate in management issues was an interesting result to emerge from this work. None of the participants were entirely dismissive of scientific research as a knowledge basis, but many felt that ‘experts’ often disregard on-the-ground knowledge and thereby come to the wrong conclusions. This has similarities to the recent Moorland Forum Understanding Predation Report (Ainsworth et al. 2016), which found that local stakeholders may be more concerned with small-scale on-the-ground knowledge, whereas scientific knowledge may be more concerned with landscape-scale management. The transformation for wildlife factor had a particularly interesting approach to research, as they felt that scientists all have their own opinions and are never going to agree, so why should land managers bother to wait for them to come to a conclusion. This highlights that scientific agreement is important for public trust in experts, which is particularly key in the current era of ‘fake news’. Being open about current debates can be beneficial (Wyborn et al. 2020), but clearly it can also have negative impacts.

Figure 3 highlights areas where more research is needed on certain management action consequences. Where there is disagreement between factors and no academic consensus, this highlights topics for future work, e.g. the effect of grip blocking on heather beetle. Figure 3 also emphasises areas where there is still debate between academics, such as the role of managed burning in carbon storage and wildfire prevention (Davies et al. 2016). Disagreement in science is a challenge for decision-makers, who do not have clear guidance for policy. There is not an easy solution to this, as scientific debate is important, but perhaps promoting areas of agreement alongside areas of difference could encourage trust.

### Wild birdlife is a point of agreement

Areas of agreement are an important result to emerge from Q-method analysis, as shifting dialogues to engage in these consensus topics can promote positive change (Carmenta et al. 2017). All three factors rated S10 and S11, which refer to impacts on wild birdlife, as important. Many participants across all factors expressed an appreciation of wild birdlife. S10 ‘Negatively impact wading bird populations (e.g. dunlin, golden plover, and curlew)’ picked up on concerns around changing management to reduce heather cover causing a decrease in nesting birds, whilst S11 ‘Benefit songbirds (e.g. skylark, meadow pipit, ring ouzel)’ picked up on the more positive aspects of changing management creating a diversity of habitats and food availability for wild birds. S10 was rated higher by the field sports management factor, whilst S11 was rated higher by factors 2 and 4. Some of the gamekeepers made the point that curlew, for example, need heather cover to nest in, but ‘don’t nest in deep heather’; heather management is therefore essential to ensure that heather cover of the right age and height is available. A few participants cited studies showing increases or decreases in certain bird species, variously attributed by participants to either traditional management or MPCW. It seems likely that a shift towards promoting wild birds in management approaches could increase agreement between different stakeholders, although care should be taken in providing objective evidence of the efficacy of diverse approaches.

### Relational values

Our thematic analysis of participant quotes highlighted the relational values participants held (Table 2). Most of these (sense of ownership, aesthetics, and stewardship) are shared between all factors, suggesting that the importance of relational values is consistent amongst diverse groups of stakeholders. These relational values interact with instrumental values, but are often seen as less valid due to their intangible nature, and so may be less frequently openly expressed.

A sense of **ownership** of the land was important across **all factors**, even in cases where the participants as individuals were not landowners. In some cases, ownership values were a result of long-term engagement with the land. This ownership was sometimes expressed as participants wanting to manage ‘their’ land for their own personal benefit. Ownership was also sometimes expressed as an exclusion of others. A sense of ownership formed a large part of individuals’ connection to the land and was consistent across all factors.

The **aesthetics** of the landscape were also mentioned within **all factors**, although the preferred appearance of the land varied. When S38 ‘Create a more beautiful landscape’ was explicitly discussed, many participants were reluctant to express an opinion, with comments such as ‘beauty is in the eye of the beholder’ ~ gamekeeper, and ‘beauty is quite subjective’ ~ employee. Comments on landscape aesthetics were, however, expressed in response to other statements. Some participants expressed a love of the landscape as it is, whilst others spoke about ambivalent or negative emotions evoked by changing appearances. Finally, some participants shared their wishes for the aesthetics of the landscape in the future. Aesthetic values therefore seem to form an unacknowledged, but nevertheless important, part of stakeholders’ values in relation to land management.

Many participants, across **all factors**, expressed a sense of **stewardship**. What that meant in practice, however, varied. Some participants spoke about maintaining traditional management, whilst others talked about the importance of change. This sense of stewardship had an interesting relationship to sense of ownership, as it seemed to evoke a longer-term acknowledgement of custodianship rather than possession. Several people talked about wanting to ‘leave it better than we acquired it’ ~ farmer. This is similar to Mould et al.’s (2020) recent study on relational values in the context of river management, where all participants felt a sense of stewardship, but had different visions of the future of the river associated with their baseline assumptions of how the river should be. In our study, individuals across all factors expressed pride in doing the right thing for the land, but did not agree on what the ‘right thing’ was.

The fourth relational value we found, a **fear of loss or wasted resources**, was not part of all worldviews, but was associated with the traditional management and farming factors (1 and 3). This value can be seen as a fear of loss of the other relational values identified in this study. A sense of ownership can be eroded when external decision-makers reduce autonomy in land management. Aesthetic values, and other values associated with finding joy in the landscape (e.g. hearing the curlews), could be diminished if the landscape appearance or wildlife changes. A sense of stewardship may be lost when stakeholders are forced to manage the land in ways that do not seem right to them. This is particularly evident in the fear of wasting the land, for example, one farmer had a particular dislike of trees being planted on farmland. Finally, there was a fear of losing a way of life, as participants wanted to maintain their identity as people who have a particular relationship with the land. These fears may be part of the reason that some stakeholders are reluctant to engage with MPCW.

### Problems with the top-down approach to land management

Many of the participants across the factor groupings agreed that the top-down approach to land management is unhelpful. This can be split into several related concerns: firstly, that people are losing their sense of control over the lands they and their forebears have managed; secondly, that the authorities are incompetent and unaware of what is best for the land; and thirdly, that decision-makers’ agendas change rapidly and do not consider long-term impacts. Some people felt that traditional management is best for conservation, but that authorities rarely recognise this. This has parallels with Soliva et al.’s (2008) findings across upland landscapes in Europe. In the Yorkshire Dales, the relationship with Natural England (NE) in particular was mentioned as problematic. Several participants stated that top-down decisions rarely take account of local knowledge, and some participants felt that decision-makers were ‘cherry-picking issues’ (estate manager) without considering the holistic long-term consequences. Schemes to change land management are likely to fail if they do not take account of local populations’ views (Bennett 2016). This is partly because local land managers have valuable insights into their own area, which incoming authorities may lack, and partly because conservation schemes often require monitoring and upkeep, and excluded stakeholders have no incentive to provide these services.

These results demonstrate that respecting local knowledge is important in the English uplands, as it is across the globe. Current conservation efforts in countries with a colonial legacy are often criticised for ignoring local knowledge, or even for criminalising the traditional management practices of local communities (Apostolopoulou et al. 2021). One estate manager expressed parallel feelings, saying there was an ‘institutional bias against people’ where the ‘practitioner has no value’. Although people in the English uplands are not currently being forced off the landscapes managed by their forebears through legislation, some feel that they may have to leave if their current livelihoods become economically unviable. Others feel that, although they are able to stay on the land, the top-down control of land management is effectively removing their sense of ownership. Changing management does not automatically mean the disenfranchisement of local communities if it is done with local stakeholder involvement. Engaging stakeholders with changing management in a way that maintains their valued relationship to the land, and building relationships of trust between stakeholders and decision-makers, are necessary and important steps. Reed (Reed et al. 2013) has developed methods for engaging with stakeholders in the British uplands, and our results reinforce the importance of employing these in conservation schemes.

## Conclusions

The four viewpoints explored in this study are more varied than simply for or against changes to peatland management. We determined that most stakeholders agree on the aims of peatland management to reduce flooding, limit wildfires, and improve habitats for wild birds, but there are differences of opinion concerning the best methods to achieve these aims. The complexities of the ecosystem and the range of interventions covered by the blanket term MPCW mean that the same actions are seen to have different consequences by different people in different places. Where the academic evidence base for management interventions is strong, clear communication of current understandings could encourage engagement. Where the academic evidence base is weak, research should be co-created with stakeholders to apply local knowledge. Areas of consensus can be considered as entry points for productive discussion in future dialogue.

Our work on relational values provides a new perspective on stakeholder engagement with peatland conservation, showing that people feel a sense of ownership towards the land, alongside valuing its appearance, and taking pride in managing it in the right way. Some land managers have concerns that MPCW may cause them to lose their relational values to the land, and these fears should be considered to allow productive discussion. We have demonstrated that top-down approaches exclude local stakeholders, and that disregarding local knowledge leads to negative impacts for both parties. Future approaches to changing peatland management should therefore include local land manager perspectives in intervention planning.

## Supplementary Information

Below is the link to the electronic supplementary material.

Supplementary file1 (PDF 220 KB)
