# Supplementary material for: Protecting peatlands requires understanding stakeholder perceptions and relational values: A case study of peatlands in the Yorkshire Dales
Source: Ambio. 2023 Apr 23;52(7):1282–96. doi: 10.1007/s13280-023-01850-3 (PMC10122872; doi:10.1007/s13280-023-01850-3)
Supplement: Supplementary file 1 — Supplementary file1 (PDF 220 KB) [file 13280_2023_1850_MOESM1_ESM.pdf]

Ambio

Supplementary Information

This supplementary information has not been peer reviewed.

Title: **Protecting peatlands requires understanding stakeholder perceptions and relational values: A case study of peatlands in the Yorkshire Dales**

Authors: Kirsten J. Lees, Rachel Carmenta, Ian Condliffe, Anne Gray, Lyndon Marquis, Timothy M. Lenton

Scenario and statements as sent to participants

Please note that this scenario has been written to simplify the likely changes that managing for carbon and water would involve. We are aware that there is still much debate around the best ways to approach these management aims. We have written this scenario to try to reflect the changes which may be encouraged under new land management incentive schemes.

Managing peatland for carbon and water aims to restore the peatland to a state in which carbon is taken in by the vegetation and stored as it decomposes into peat, and rainwater moves slowly through the landscape and is naturally filtered before reaching watercourses. The ideal peatland under this scenario has high water levels all year round, and a mix of vegetation including Sphagnum moss, dwarf shrubs such as heather, and sedges. The peat surface should be wet enough that kneeling or sitting on it gets clothes wet immediately. Managing peatland for carbon and water involves a variety of approaches. These can include blocking grips and gullies to raise the water table, leaky dams on watercourses to slow run-off, stabilizing and revegetating bare peat to reduce erosion, and altering the mix of plants present. Frequent burning is unlikely to be compatible with managing for carbon and water, although heather cutting may be. Grazing can be used as a management method to limit dominance of a single vegetation species, and should be at stocking densities which allow healthy mixed vegetation to grow.

The statements below are suggestions of what the results of this management might be. They are statements of perception, not necessarily scientific facts, and we are interested in what you personally believe to be most or least important when deciding how to manage the land.

Please sort the statements into 'important to me' and 'not important to me' groups, based on your perception of what the above scenario would cause to happen.

Managing peatland for carbon and water would…

1.Increase risks to livestock, eg. Liver fluke, foot rot, and poisonous plants such as bog asphodel
2.Lower sheep stocking rates on moorland to economically unviable levels
3.Increase stocking rates on inbye or allotments
4.Cause livestock to drown in wetter areas
5.Prevent lambs being lost in grips
6.Increase crane flies and larvae for young birds to eat
7.Increase vegetation cover on bare peat areas
8.Reduce heather dominance
9.Increase rare vegetation such as Sphagnum moss and sundew
10.Negatively impact wading bird populations (eg dunlin, golden plover, and curlew)
11.Benefit songbirds (eg skylark, meadow pipit, ring ouzel)
12.Increase ticks and associated illnesses
13.Increase heather beetle
14.Cause vehicles to be bogged more often
15.Reduce wildfire spread due to wetter ground

16. Increase wildfire severity due to higher fuel loads
17. Increase local flash flooding
18. Mitigate flooding downstream
19. Reduce soil erosion
20. Reduce reservoir sedimentation
21. Help to provide clean, clear water
22. Make water treatment cheaper
23. Reduce dog walkers straying off paths
24. Require training in new skills
25. Need to involve external experts to plan management
26. Only work on long timescales/need guaranteed long-term funding
27. Increase carbon capture to help meet net zero by 2050
28. Detract from greater management considerations
29. Be a waste of taxpayers' money
30. Need to rely on external funding
31. Involve too much bureaucracy
32. Affect the tradition of hefted sheep
33. Affect farmers' identity as food producers
34. Make all the work that's previously been done on the land pointless
35. Reduce my control of the landscape
36. Strengthen my custodianship of the landscape
37. Affect commoners' rights
38. Create a more beautiful landscape
39. Make irreversible changes to the landscape
40. Make me miss the landscape of my youth
41. Move away from being a traditional cultural working landscape
42. Require better scientific consensus on issues
43. Increase maintenance needed on rights of way
44. Cause trees to self-seed on the moorland
45. Increase nature tourism
46. Create unforeseen new problems
47. Make driven grouse shooting economically unviable
48. Improve the public image of moorland management
49. Reduce air pollution from managed burns
50. Provide new local employment opportunities in restoration
51. Reduce my traditional income source
52. Provide a potential new income source
53. Not involve local workers
54. Need to be promoted by people/organizations I trust

Standard Operating Procedure

1. Participant recruitment

Participants will be recruited via project partner networks and via social media advertisements. Anyone self-identifying as a land manager on peatland in the Yorkshire Dales over the age of 18 can be included.

All interested people will be directed to contact K Lees, who will collect personal information for contact purposes (name, email, address, phone number if necessary) and determine whether implementing the method via zoom call will be possible. The information sheet and consent form will also be emailed to the participant to be signed and returned via email where possible. A convenient time for the first call will be arranged.

2. Participant pack

A participant pack will be sent, containing:

- Letter and list of contents
- Scenario and pre-cut Q-statements in an envelope labelled Q-method
- Pre-cut grid
- *Information sheet and consent form, and stamped addressed envelope, if the participant is not able to sign and return via email.*
- Packet of biscuits

3. Initial call

This will be a short conversation to explain the method and collect some basic demographic information. This will also serve as a test of the zoom connection! The researcher will:

- Explain the aims of the study and highlight that we are interested in the opinions of local people, not the facts.
- Answer any questions about the information sheet and ask the participant to sign and return the consent form if they haven't already.
- Collect some basic demographic data: gender, age bracket, land manager role, and whether they have any engagement with peatland restoration schemes.
- Read scenario and ask the participant to sort the statements into important/unimportant groups in their own time, and take a photo/record statement numbers.
- Arrange a time for the second call if possible, and encourage the participant to keep 2 hours free of distractions as much as possible.

After the call, send an email reminding the participant to send a photo of their initial sort into bins, and confirming the time of the second call.

4. Second call and Q-sort

This may be a group call with several participants and researchers present. If this is the case then researchers and participants will be paired. The Q-method will be explained to the group and the Q-

sort initiated – implementing this during the group call will hopefully reduce the pressure on the individual participants, but each researcher should take note of relevant comments made by their paired participant.

It is a good idea for the researchers to have a copy of the grid and the statements handy, so that they can explain the structure to the participants and cross-reference statements and numbers.

The researchers and participants will then be split into breakout rooms for follow-up questions. Each researcher should ensure that their participant's Q-sort is recorded, either by making notes during participation or by asking the participant to send a photo or record of statement numbers. Follow-up questions should be asked about the five top and bottom statements, and may include:

- What do you think about …., why did you place this card in this position?
- Prompt them to talk about their experiences, or those that they know about.
- If specific actors are involved/affected, ask participants to specify in greater detail who they feel is involved/affected.
- If people need a prompt to help them think of responses add: For example, have you, your family or neighbours had an experience with this? Or have you heard something about this that influenced your opinion? What kind of information?
- Jot down direct quotes in speech marks.
- If people are unclear, try to discuss the statement with them until it becomes clear.
- Ask about 'paired' statements.

The participants and researchers will then return to the group, we will thank participants and explain that we will send a short report when we have some results.

5. Data recording

Participant datasets (except identifying data - name, address, email and phone number) will be stored in Excel files, and the files will be named A, B, C etc. to ensure that they are anonymous. Within each Excel file four sheets will be used:

1. The data gathered on role, gender, age, and interaction with restoration schemes.
2. The initial sort of statements into bins.
3. The Q-sort.
4. The reasons given for the statements in extreme positions.

6. Follow-up

A voucher will be sent to all participants, via email where possible, to thank them for their time.

After the Q-method is completed and the results analysed, a short report will be sent to all participants.

Table S1 – statements that were not considered very important in any factor, the reasons why, and an example quote.

Statement	Reasoning	Example quotes
S4 'Cause livestock to drown in wetter areas'	Most factors rated livestock issues as of minimal concern. The farming factor stated that restoration wouldn't impact this issue.	'happens anyway' ~farmer
S5 'Prevent lambs being lost in grips'	This was seen as a beneficial side-effect, but not something that happened often enough currently that it would affect decision-making.	'never seen it' ~gamekeeper
S12 'Increase ticks and associated illnesses'	Most participants believed that ticks were not a problem in their area.	'quite lucky here - we don't have a tick problem' ~gamekeeper
S14 'Cause vehicles to be bogged more often'	This was seen as an unavoidable part of life on a peatland area.	'shouldn't be driving on blanket bog anyway. If you are you make provision for it.' ~estate manager.
S23 'Reduce dog walkers straying off paths'	Opinion was split, with some participants not considering this an issue, and others believing that restoration would not cause it to change.	'vast majority walk elsewhere or on a lead' ~gamekeeper 'don't believe that for one minute…[the] whole of the Yorkshire Dales is a dog park' ~farmer
S28 'Detract from greater management considerations'	All factors felt that there was no greater consideration than land management.	'don't think there are many more issues more important than upland management round here' ~farmer
S32 'Affect the tradition of hefted sheep'	Participants stated that most sheep are no longer hefted anyway.	'don't think this romanticised James Herriot working landscape exists anymore' ~employee
S34 'Make all the work that's previously been done on the land pointless'	Some people felt that changing management would not obscure what had gone before. Others felt that previous work was not a good idea originally.	'why did we grip the moors? Why did they not think that's going to increase flooding?' ~gamekeeper
S37 'Affect commoners' rights'	All factors said that commoners' rights are not an issue in their areas.	'doesn't affect us' ~gamekeeper
S40 'Make me miss the landscape of my youth'	Most participants were quite open to the idea of change, so long as it accorded with their ideas.	'no objection to change' ~gamekeeper 'maybe some people did like walking round the peak district and coming back black full of coal dust' ~employee

S45 ‘Increase nature tourism‘	This was widely considered to be an unlikely source of income, or a bad idea.	‘nature's out there for everyone to enjoy - we don't own it’ ~farmer<br>‘these areas are attractive with or without’ ~estate manager
